# Validity and Reliability of the Greek Version of Adult Vaccine Hesitancy Scale in Terms of Dispositional Optimism in a Community-Dwelling Population: A Cross-Sectional Study

**DOI:** 10.3390/healthcare12151460

**Published:** 2024-07-23

**Authors:** Marilena Gialama, Christos Kleisiaris, Maria Malliarou, Dimitrios Papagiannis, Ioanna V. Papathanasiou, Savvato Karavasileiadou, Wafa Hamad Almegewly, Konstantinos Tsaras

**Affiliations:** 1 Department of Nursing, School of Health Sciences, University of Thessaly, 41500 Larissa, Greece; mgialama@uth.gr (M.G.); kleisiaris@uth.gr (C.K.); malliarou@uth.gr (M.M.); dpapajohn@uth.gr (D.P.); iopapathanasiou@uth.gr (I.V.P.); ktsa@uth.gr (K.T.); 2 Department of Community and Psychiatric Mental Health Nursing, College of Nursing, Princess Nourah bint Abdulrahman University, Riyadh 11671, Saudi Arabia; skaravasileiadou@pnu.edu.sa

**Keywords:** vaccination, vaccine hesitancy, adult Vaccine Hesitancy Scale, COVID-19, validity, factor analysis, reliability, determinants, Greece

## Abstract

Vaccine hesitancy is an important public health issue referring to concerns about the safety and efficacy of vaccination. Within a framework, this study aimed to assess the cultural adaptation, validity, and reliability of the Greek version of the adult Vaccine Hesitancy Scale (aVHS) as well as to identify the determinants of vaccine hesitancy among a large regional population in central Greece. A cross-sectional study was conducted enrolling 300 adults who had received primary healthcare services in the Health Centers and Local Health Units of the Magnesia Region from October to December 2022. The aVHS and the Life Orientation Test-Revised (LOT-R) were used to identify vaccine hesitancy and the dispositional level of optimism, respectively. For survey translation, the procedure of forward and backward translation was followed. Also, the aVHS was tested in a pilot study with a sample of 18 responders. Construct validity and internal consistency reliability were investigated via exploratory and confirmatory factor analysis and Cronbach’s alpha coefficients, respectively. Simple and multiple linear regression analysis were used to determine predictors for vaccine hesitancy. Factor analyses indicated that the aVHS comprises two constructs (“lack of confidence” and “risk perception”) explaining 68.9% of the total variance. The Cronbach’s alpha of the total scale was 0.884, indicating its high internal consistency. Participants who lived in rural areas, had a lower annual income, and reported a lower level of optimism showed a higher lack of confidence in vaccination. On the other hand, people aged above 45 years old who had graduated from high school or elementary school and were unemployed showed greater aversion to the risks of side effects. Finally, certain socio-demographic characteristics were associated with vaccine hesitancy. Our data suggest that the aVHS is a valid and reliable instrument for measuring vaccine-related attitudes and perceptions in Greek society, providing meaningful insight into designing vaccination-related preventive interventions in the community.

## 1. Introduction

The World Health Organization (WHO) has identified vaccine hesitancy as one of the top ten threats to global health, which can potentially jeopardize progress in the fight against diseases that can be prevented through vaccination [1]. Specifically, the term “vaccine hesitancy” (VH) is used to describe a range of behaviors, from mild reluctance to outright refusal [2]. The Strategic Advisory Group of Experts (SAGE) Working Group defines VH as “the reluctance or delay in receiving a vaccination despite the availability of vaccination services” [3]. VH varies over time, location, and vaccines. Moreover, it presents a certain degree of complexity while it remains context-specific [4].

It has been suggested that complacency, convenience, and confidence influence the construction of the 3Cs model of VH [4,5]. Complacency is based on the reduced perception of disease risk brought on by the infection’s uncommon occurrence. Confidence is the belief in the efficacy and safety of vaccines and the confidence in decision makers who make vaccination recommendations. In addition to economic and geographic considerations, convenience also relates to the comfort and quality of the service, as well as the ability of the public to comprehend the importance placed on immunization [5,6].

Although the majority of communities worldwide accept vaccination as a standard practice, a smaller proportion exhibit selective acceptance, opposing certain vaccines while accepting others [4,7]. Regarding hesitancy for COVID-19 vaccination, its multifaced nature involves various demographic, psychological, and social determinants. In particular, recent studies have shown that younger people, women, and those with lower educational levels are more likely to be vaccine hesitant. This hesitancy often derives from concerns about vaccine safety and efficacy, perceived low risk of contracting the disease, and the belief that the disease is relatively mild [8,9]. Moreover, lack of trust in the healthcare system and exposure to misinformation and conspiracy theories intensify VH [10,11]. In addition, socioeconomic factors such as higher educational attainment and monthly income are associated with lower VH and higher vaccination coverage [12]. A thorough understanding of these determinants is critical to measuring VH in a community and developing targeted public health interventions aimed at increasing vaccine uptake and improving overall public health outcomes.

A new dimension to consider is that VH has been significantly associated with an individual’s level of dispositional optimism, especially concerning perceived risks associated with vaccination [13]. In general, dispositional optimism (expectancies regarding future outcomes) is linked to proactive health behaviors, including regular medical check-ups, healthy eating, and exercise, as optimists are more likely to engage in behaviors that prevent illness and promote long-term health [14,15]. Studies have demonstrated that higher dispositional optimism is associated with better psychological resilience and coping strategies during health crises [16].

In Greece, the polarization of the community on the subject of vaccines during the pandemic seemed to be the ideal ground to investigate VH. Despite 76.12% of the Greek population having received at least one dose of the COVID-19 vaccine [17], a substantial proportion remains unvaccinated. Also, the trends in vaccination intention against COVID-19 and associated determinants in the general population have been well documented in several studies, highlighting the need for consistent and accurate information to address VH [8,12,18]. Nevertheless, to our best knowledge, there are no studies available on VH using a validated measuring tool in the community-dwelling population.

Therefore, we hypothesized that a more comprehensive investigation of VH within the Greek community will yield valuable insights for community health. Considering that the pandemic significantly influenced individuals’ beliefs and attitudes toward vaccination, understanding these dynamics is crucial. The COVID-19 pandemic was a pivotal period that shaped public perceptions and could potentially impact future vaccine adherence. Within a framework, aVHS will help in developing targeted interventions and policies to enhance vaccine uptake and ensure sustained public health benefits.

Consequently, this study aimed to evaluate the cross-cultural adaptation, validity, and reliability of the Greek version of the aVHS in the context of individuals’ thoughts, feelings, attitudes, and actions among a sizeable regional population in Greece.

## 2. Materials and Methods

### 2.1. Study Design and Participants

A cross-sectional correlational study was performed involving 300 community-dwelling people in the Magnesia region in central Greece. A convenience-sampling method was applied to recruit participants who visited the local primary healthcare settings in the region of Magnesia—a province of Thessaly—where the University of Thessaly is located.

According to the literature, a minimum sample size for the exploratory factor analysis (EFA) of 50 observations [19] and the confirmatory factor analysis (CFA) of 200 observations was required [20]. Thus, the sample size for the precision of the study was calculated with the G* Power 3 software. Considering a low effect size (f^2^ = 0.04), the precision level of 5% (alpha level), the statistical power of 90%, and the total number of predictors as 10, a minimum sample size of 265 individuals was required. To further reduce random error in our results, we recruited more participants by distributing a total of 350 questionnaires (85.7% response rate).

Given that 76.12% of the Greek population has received at least one dose of the COVID-19 vaccine [17], by selecting participants who have received at least one dose, we ensured that they had already overcome initial barriers, allowing us to focus on VH itself rather than preliminary factors like access to the vaccine and fear of needles. Taking all the above into consideration, the criteria included the following: (1) individuals aged 18 years or older, (2) having received at least one COVID-19 vaccination, and (3) fluency in the Greek language. Exclusion criteria encompassed the following: (1) cognitive impairment, (2) a documented history of mental illness, and (3) refusal to provide informed consent for participation in the study and complete the questionnaire.

### 2.2. Data Collection and Measures

The data were collected in primary healthcare settings (Health Centers and Local Health Units) using a self-reporting questionnaire. The data collection tool consists of two parts. In the first part, various socio-demographic characteristics of the participants and the Life Orientation Test-Revised (LOT-R) are questioned. In the second part, there is the 10-item form of the aVHS. The administration of the questionnaires was conducted by the main researcher (M.G.). We recruited those individuals who visited local healthcare facilities to receive primary healthcare services. Their written consent was obtained before collecting the required data. The survey was conducted between 20 October and 20 December 2022.

#### 2.2.1. Adult Vaccine Hesitancy Scale (aVHS)

The aVHS was created to provide a measure pertinent to the vaccination-related views that the general population of adults holds. The VHS, originally developed by researchers to investigate parents’ attitudes and beliefs, was the basis for its development.

Larson et al. created this scale to examine vaccination-related reluctance, attitudes, and issues in a population of parents [21]. In their study, Shapiro et al. validated the nine-item VHS Likert-type scale using a population of parents. Ten items were initially included, but the researchers excluded them due to the lack of statistical reliability [22]. The English version consists of two dimensions: “lack of confidence” and “risk perception” or “risks” [22].

In a recent study, Luyten et al. modified the wording of the VHS items, excluding any reference to children and parenthood, to make it applicable to the general population [13]. Their scale adaptation was used as the basis for developing the Greek version of the aVHS.

This aVHS version includes ten items. The five-point Likert scale, which ranges from 1 (“strongly disagree”) to 5 (“strongly agree”), shows how each item is answered. Seven questions (items 1, 2, 3, 4, 6, 7, and 8) are scored in reverse order. Higher aVHS scores indicate a greater level of VH.

##### Cultural Adaptation of the Greek Version of aVHS

We adhered to the Sousa and Rojjanasrirat guidelines for the cross-cultural adaption of research instruments using the modified VHS for the adult population [23]. Permission to use, translate, and validate the psychometric properties of the aVHS scale in Greek was acquired from the developer of the scale.

Forward translations of the aVHS were developed by two native Greek speakers with backgrounds in nursing and public health who translated the English version of the aVHS. The translation process aimed more at maintaining the context of the questionnaire rather than providing a translation derived from the exact words of the initial text. Moreover, translators adapted the vocabulary and the expressions to Greek contexts and settings, avoiding, at the same time, the use of jargon or formal language. A third independent translator compared the two forward translations that were developed and evaluated them regarding ambiguities, meanings, sentences, and terminology.

The reverse translation of the scale version was created by two bilingual translators. One of them was employed in the medical field, while both were unaware of the initial questionnaire. A comparison of the scale’s original and back-translated forms was made, with a focus on any terminology or components that would be especially vulnerable to distortion during translation or that are essential to the instrument’s subject.

The two back-translated versions of the aVHS were compared and evaluated by a multidisciplinary committee to resolve any ambiguities or discrepancies within the wording of the instrument. The pre-final version of the aVHS in the Greek language underwent a pilot testing process after the committee granted the instrument its approval.

**Figure 1:**
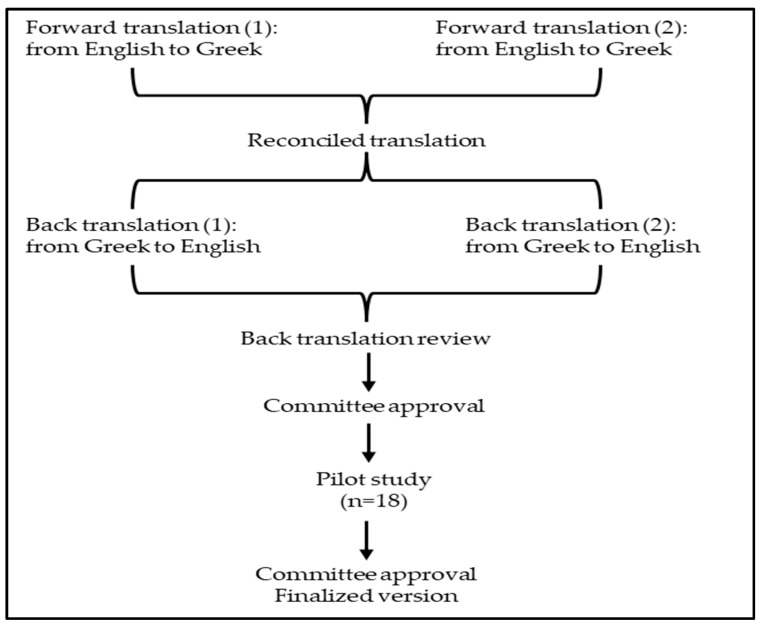
Cross-cultural adaptation of the Greek version of the adult Vaccine Hesitancy Scale (aVHS).

The scale was tested on a sample of 18 responders with a range of gender, age, and educational backgrounds. Participants were provided with paper forms that included additional spaces for feedback on aspects related to cognitive interviewing, such as their thought processes when selecting a particular response to a scale item, the rationale behind their choices, and any phrases or expressions they found unclear. The changes suggested by the participants were discussed among the members of the committee that was in charge of the transcultural adaptation, to determine whether or not the questions’ context was preserved. After incorporating all the proposed modifications, minor adjustments were made, resulting in the creation of the final version of the questionnaire (Figure 1).

#### 2.2.2. Assessment of the Dispositional Optimism

To investigate whether more optimistic individuals were more likely to adhere to vaccination, we assessed the participants’ optimistic or pessimistic expectations (thoughts, feelings, attitudes, and actions) about the future using the Greek version of the LOT-R questionnaire [24]. Specifically, the LOT-R is a short instrument for assessing dispositional optimism [25]. The instrument comprises ten items, six of which are used to assess the levels of optimism (3 items) or pessimism (3 items) and are referred to as “core items” (for example “*In uncertain times, I usually expect the best*”, “*If something can go wrong for me, it will*”, etc.), and the remaining four items are supplementary questions included to conceal the primary purpose of the test and are referred as “filler items” (for example “*It’s easy for me to relax*”, etc.). Participants indicated the extent to which they agreed with each of the items on a 5-point scale from 0 (“strongly disagree”) to 4 (“strongly agree”). The total score for the LOT-R is derived by summing the scores for the six core items (ranging from 0 to 24) after reversing the pessimism items. Higher scores indicate higher levels of dispositional optimism.

The demographic (gender, age, marital status, having children and their age), socioeconomic (place of residence, level of education, employment status, individual annual income) characteristics, and self-perceived health status of the participants were also recorded.

### 2.3. Ethics

This study is a part of the Nursing Laboratory (“Public Health & Vaccines Laboratory”) public health activities in community healthcare and therefore meets ethical approval standards. In particular, the study protocol was approved by the Institutional Review Board of the Nursing Department, University of Thessaly (decision No. 22/13-07-2022) and the Ethics Committee of the 5th Regional Health Authority of Thessaly & Sterea of the Ministry of Health (decision No. 46447/08-06-2021). All participants were given explanations about the aim of the study and signed an informed consent form. The research was conducted following the principles of confidentiality and anonymity.

### 2.4. Data Analysis

Quantitative variables are reported with mean, standard deviation, and range (minimum–maximum). Qualitative variables are summarized as absolute (*n*) and relative (*%*) frequencies. Kolmogorov–Smirnov test and normal Q–Q plots were used to estimate the normal distribution of quantitative variables. EFA was conducted on the construction set to evaluate the construct validity of the aVHS. Principal component analysis was chosen as an extraction method using varimax rotation. The cut-off point for factor loadings was 0.40 and for eigenvalues 1.00. Also, a CFA was carried out on the same set to examine the goodness of fit of the model resulting from the EFA, using several absolute fit indices [chi-squared/degrees of freedom (*χ*^2^/*DF*), root-mean-square error of approximation (RMSEA), standardized root-mean-square residual (SRMR), goodness-of-fit index (GFI)] and incremental fit indices [normed fit index (NFI), Tucker and Lewis index (TLI), relative fit index (RFI), comparative fit index (CFI)]. Based on the literature [13,20,22,26], recommendations regarding the expected coefficient values for acceptable model fit were as follows: for (*χ*^2^/*DF*) ≤ 3; for RMSEA ≤ 0.08; for SRMR ≤ 0.08; for GFI ≥ 0.90; for NFI ≥ 0.90; for TLI ≥ 0.90; for RFI ≥ 0.90; for CFI ≥ 0.90. The internal consistency was appreciated using Cronbach’s alpha (*α*) coefficient for testing the reliability of the aVHS. A Cronbach’s alpha cut-off of 0.70 indicates adequate internal consistency and acceptable reliability of the scale [20]. To explore the association of socio-demographic characteristics and the LOT-R score with the scores in the dimensions of aVHS, as a result of factor analysis, univariate and multivariate linear regression analysis in a stepwise method were used. Regression coefficients (*β*) with standard errors (*SE*) were reported from the linear regression analyses. Statistical significance was set at the 0.05 level (2-tailed). Data were analyzed using IBM SPSS 26.0 and JASP 0.17.2.

## 3. Results

### 3.1. Participant Characteristics

The mean age of 300 adults (110 men and 190 women) was 44.87 (*SD* = 17.24; ranging from 20 to 84) and aged between 20 and 29 years (28%), 50 and 59 years (22%), and over 60 years (21.7%) across age groups. Overall, the majority of participants were married (50%), had children (57.3%), lived in urban areas (74.7%), were university graduates (54%), and were currently working (58%). Regarding the optimistic expectations for the future, the mean overall LOT-R score was 2.32 (*SD* = 0.64, ranging from 0.33 to 4.00). The detailed socio-demographic characteristics of the sample are presented in Table 1.

### 3.2. Vaccine Hesitancy

The aVHS investigates certain aspects that are responsible for developing resilient vaccine skepticism and eventually VH. Four out of five participants agreed that vaccines are important for their health. Most of the participants considered vaccines effective (79.7%) and vaccination important for the health of others in the community (86.0%). Additionally, a great percentage (36.3%) seem to either disagree or be uncertain about whether all routine vaccinations recommended by the government are beneficial. The results presented a similar image when the participants were asked whether they believed that the vaccines offered by the government are trustworthy, more specifically 9.6% disagreed with that statement and 38.0% were unsure. Also, the vast majority of the population agreed that vaccines are a good way to protect someone from diseases (81.0%) and follow the recommendations of doctors or healthcare providers regarding vaccines (77.0%). The results show that almost half of the participants are uncertain about whether the new vaccines are more dangerous than older vaccines, and a significant percentage (56.0%) of participants seem to be concerned about the adverse effects of vaccines. Lastly, the greatest part of the participants (53.7%) either disagree or are uncertain about the necessity of vaccination for diseases that are not common anymore (see Table 2).

### 3.3. Construct Validity of the aVHS

Exploratory and confirmatory factor analysis methods were used to evaluate the construct validity of the aVHS (see Table 3). Principal component analysis was performed, applying the Kaiser–Guttman criterion for selecting factors (eigenvalues > 1.00). Also, a varimax rotation was chosen to assist with the interpretation of the findings. Before performing the principal component analysis, the suitability of the data for factor analysis was evaluated. The value of the Kaiser–Meyer–Olkin test was 0.897 (above the limit of 0.60); thus, the sample size was adequate to conduct the EFA. Furthermore, Bartlett’s test of sphericity was significant and confirmed the correlation matrix’s factorability (*χ*^2^ = 1760.84, *p* < 0.001). In addition, the suitability of each item of the aVHS was measured with anti-image correlation. Anti-image correlation values of all items ranged from 0.779 to 0.935 (above the limit of 0.50). For these reasons, the aVHS data set was found to be appropriate for factor analysis.

Items loadings as produced from EFA are presented in Table 3. In the final model, all items were entered into the factor analysis. All items’ loadings in factors had values >0.40 (ranging from 0.692 to 0.857), which is the cut-off point for acceptance. Moreover, none of the items had loadings above 0.45 in more than one factor, as has been suggested by several researchers. In line with this, two factors emerged that interpreted 68.9% of the total variance. Their initial eigenvalues were 5.253 for the first factor and 1.637 for the second factor, while after rotation they were 4.690 and 2.200, respectively. The first factor consisted of seven items of the scale and explained 52.5% of the variance (46.9% after rotation). The second factor included the remaining three items and explained 16.4% of the variance (22.0% after rotation). The two-factor structure included the factors “lack of confidence” (Factor 1) and “risk perception” (Factor 2) analogically to the factor names of the original parental VHS [13]. Also, CFA was performed to examine the model fit resulting from the EFA. The fit indexes for the two-dimensional model, including all ten items of the scale, clearly showed a good fit. The model fit indices were as follows: (*χ*^2^/*DF*) = 3.1, RMSEA = 0.084, SRMR = 0.038, GFI = 0.996, NFI = 0.936, TLI = 0.939, RFI = 0.916, and CFI = 0.954.

### 3.4. Internal Consistency Reliability of the aVHS

The mean, standard deviation (*SD*), and range of the items in each aVHS factor are shown in Table 4. On a scale from 1 to 5 (with 5 being maximal hesitancy), the mean overall aVHS score was 2.30 (*SD* = 0.60, ranging from 1.00 to 4.00). The mean scores for the “lack of confidence” factor and “risk perception” factor were 2.03 (*SD* = 0.67, ranging from 1.00 to 4.29) and 2.94 (*SD* = 0.80, ranging from 1.00 to 5.00) respectively, which means that hesitancy in our sample was due more to risk perceptions than to lack of confidence in vaccines.

Cronbach’s alpha coefficients, the change in Cronbach’s alpha coefficient when the item was deleted, and item-total correlations were examined for aVHS reliability (see Table 4). All the scales of aVHS exceeded the minimum reliability standard of 0.70. More specifically, Cronbach’s alpha was 0.919 for the “lack of confidence” dimension and 0.776 for the “risk perception” dimension, demonstrating adequate internal consistency reliability. Cronbach’s alpha value of the overall scale was found to be highly reliable at 0.884. In this line, Cronbach’s alpha values in two subscales, in case an item was removed from the subscale, were checked. The audit showed that Cronbach’s alpha values after excluding individual items were lower for all items of the instrument. Moreover, the correlation of individual items to the total score was between 0.561 (for item 10) and 0.811 (for item 2).

### 3.5. Factors Associated with aVHS Domains

Nine socio-demographic characteristics and the life orientation score were analyzed using simple and multiple linear regression models to recognize independent variables that could predict vaccination hesitancy among community-dwelling adults. Univariate analyses revealed several variables statistically associated with aVHS dimensions (see Table 5). Factors significantly related to the “lack of confidence” subscale were place of residence, educational level, annual income, and life orientation score. Regarding the “risk perception” subscale, five variables, i.e., participants’ age, marital status, children’s age, educational level, and employment status, were significantly associated with an aversion to risks of vaccine-induced side effects.

The results of the stepwise linear regression model are shown in Table 5. For the first construct, people living in rural areas had a greater lack of confidence in vaccination than those from urban areas. In addition, participants with an annual income of EUR 10,000 to 20,000 and below EUR 10,000 showed a higher lack of confidence than participants with a yearly revenue of more than EUR 20,000. The optimistic expectations for the future were another independently associated variable with a lack of vaccine confidence. Specifically, the LOT-R score was negatively related to the lack of confidence score, which means that the lower the level of optimism, the higher the lack of confidence in vaccination. The final model, which included three variables, was statistically significant (*F* = 5.908, *p* < 0.001) and explained only 6.2% of the variance in the “lack of confidence” subscale (adjusted *R*^2^ = 0.062). Regarding the second construct of the aVHS questionnaire, participants over 45 years had greater risk perception than those under 45 years. Also, people who had graduated from high school and elementary school showed greater aversion to risks than those who had graduated from university. Finally, unemployed participants reported higher levels of risk perception than employed participants. This statistically significant (*F* = 9.941, *p* < 0.001) multivariate model, which included three variables, seems to explain 13% of the variance in the “risk perception” subscale (adjusted *R*^2^ = 0.130).

## 4. Discussion

This study aimed to validate the Greek version of the aVHS in the context of dispositional optimism in the Greek general population receiving primary healthcare services in a community. The Cronbach’s alpha of the total scale was 0.884, indicating its high internal consistency. An EFA showed that the aVHS comprises two constructs (factors): “lack of confidence” which explained 52.5% of the variance and “risk perception” which explained 16.4% of the variance. Both factors explain the percentage of 68.9% of the total variance. A CFA showed a model of two factors with ten items. We also found significant associations between VH’s factors (lack of confidence and risk perception) and the socio-demographic characteristics and the dispositional level of optimism.

VH leads to under-immunization of the population and increases the risk of vaccine-preventable diseases [22]. After assessing the cross-cultural adaption, this study examined the validity and reliability of the Greek version of the aVHS. The aVHS has been extensively tested and used in studies worldwide, and in different settings and populations, proving it is a valid psychometric instrument [13,26,27,28,29,30,31,32]. The analysis of item loadings and the validity of the questionnaire has led to the development of various versions that characterize VH differently. These versions include aspects such as adherence to complementary and alternative medicine, patient–provider interactions, and sociocultural factors. The aim is to establish the mediating effects of VH on under-immunization and to design interventions to improve vaccination communication and counseling among healthcare professionals and the general public, including parents, the elderly, chronically ill individuals, and other relevant groups. The EFA confirmed that the two-factor structure of the scale, encompassing all 10 initially proposed items, is appropriate and internally consistent in the Greek language, reaffirming the structure previously reported by other researchers. It is still debatable whether the scale should include the tenth item, “I do not need vaccines for diseases that are not common anymore”, because some studies opt to exclude it and others do not [30,32]. The two factors defined as “lack of confidence”, including items 1 to 4 and 6 to 8, and “risk perception”, including items 5, 9, and 10, were established based on the results of the EFA for the Greek version. CFA was conducted to examine the model’s goodness of fit using absolute and incremental fit indices, including chi-squared, RMSEA, SRMR, GFI, NFI, TLI, RFI, and CFI coefficients.

Another important finding was that certain socio-demographic characteristics (age, place of residence, educational level, employment status, individual annual income) and the dispositional level of optimism are associated with VH. Adults constitute a rather heterogeneous study population considering their age, educational level, employment, or place of residence. In comparison to our results, an almost similar study showed that the participants’ concerns, which increase VH, derive from religious beliefs, fears about potential side effects, or beliefs that the diseases that vaccines aim for are not harmful [33]. Studies investigating VH in other countries confirmed that VH is associated with age, employment status, educational level, and religious beliefs [33]. Income and socioeconomic status are factors reported in the bibliography that affect vaccine acceptance [34].

Our findings indicated that the age of the participants was associated with the development of VH. Particularly, people aged under 45 years old were less vaccine-hesitant considering their levels of risk perception, compared to those aged above 45 years. On the contrary, recent studies have shown that younger people were more hesitant to take the COVID-19 vaccine compared to the older age groups [35,36]. A critical reason for people avoiding COVID-19 vaccination is the proliferation of misinformation. While older adults are less inclined to obtain misinformation from online platforms compared to younger adults, they still face a risk of being exposed to inaccurate information through television or radio programs due to the widespread dissemination of COVID-19 misinformation [37]. Apart from the factor of the participants’ age, the age and the number of children that the participants had have also been investigated as potential factors for developing VH [34,38,39]. Nevertheless, the limited studies that incorporate these parameters concerning children do not indicate any significant association [37,40].

This study also showed that people who live in rural areas are more vaccine-hesitant because of their lack of confidence in the vaccination practice and the vaccines. The place of residence of the participants was a factor investigated in large-scale studies about VH. The greater availability of healthcare resources and information in urban locations can have a beneficial impact on vaccination acceptance [41]. Moreover, higher financial position and education levels are typically seen in urban settings, and these factors are linked to greater vaccine acceptability. Urban and rural communities may have different perspectives on the prevalence of diseases that vaccination can prevent [41].

Notably, although a higher level of education is strongly associated with better health status and health knowledge in general, we noticed that in certain studies, the level of schooling functioned as a barrier to the decision to vaccinate [3]. Specifically, in two studies conducted in the United Arab Emirates and Saudi Arabia, participants with higher levels of education were more skeptical towards vaccination, scoring higher on the VH scale [42,43]. There is an increasing worry on a global scale that parents with higher levels of education are more likely to seek exemptions from vaccinations because they are more likely to be skeptical [43]. In a large-scale study, higher education was found to be a potential barrier in research conducted in China, Lebanon, Israel, Bangladesh, and the USA. In contrast, it was a promoter of vaccination in studies conducted in The Netherlands, Nigeria, Pakistan, and Greece [3]. Consistent with the latter, the lower educational level of our participants was significantly associated with VH due to a lack of confidence in the vaccines and risk perception.

Furthermore, several studies support that unemployment causes individuals to be more hesitant toward vaccinations [35,44]. Specifically, unemployed people are potentially more skeptical about the vaccine’s effectiveness and safety than those with jobs [34]. Employment status may interfere with insurance status in some countries, possibly affecting people’s access to vaccines [37]. A higher probability of accessing a healthcare provider is associated with having insurance [37]. Our study showed that unemployed people reported significantly higher VH due to “risk perception” than employed participants.

We also found that lower annual income was associated with the development of VH due to a lack of confidence. Possible explanations for this association could be that participants with lower income and socioeconomic status are notably more concerned about the adverse effects of the vaccines [34,35,44]. In addition, higher socioeconomic status was also reported as a factor that leads to VH among older Argentinian people [31]. Along the same lines, marital status, level of education, and income may also play an important role in VH in Brazilians [39]. It is obvious, therefore, that financial status across all ages is degerming the action of vaccination.

In a UK study, the LOT-R scale was used to investigate the relationship between future expectations and attitudes toward vaccination [13]. The bibliography supports that the level of people’s optimism plays an important role in the decision-making process about their health and well-being [13]. In a study where the population sample consisted of nursing students from three different countries, it was demonstrated that students’ health-related behaviors were significantly predicted by their dispositional optimism [45]. Additionally, research showed that the four categories of health behaviors—healthy eating, preventative activity, positive mental attitude, and health practices—and dispositional optimism intensity were associated with each other [45]. This aligns with the results of our study, where the lack of confidence in vaccination was inversely correlated with the LOT-R score, indicating that a lower degree of optimism was associated with a higher level of lack of confidence in vaccinations.

Among other important explanatory factors reported in the bibliography was encouragement from others (e.g., friends, colleagues, or acquaintances), noting that perceptions of social and professional support towards vaccination are potentially influential in the decision of an individual to vaccinate [46]. In addition, there is a difference between the levels of VH reported by people who consult general practitioners (GPs) and the ones who consult pediatricians about vaccination [38]. It is also worth mentioning that the attitudes of the GPs or the pediatricians towards vaccines can significantly impact their patients’ vaccine behavior [47]. This introduces a new question about the way that physicians of different specialties communicate the need for vaccination to the public and influence the perspective of their patients regarding prevention and immunization strategies.

### 4.1. Future Implications

To enhance vaccination acceptance, it is essential to employ a comprehensive strategy that addresses various psychological, social, and logistical barriers. Education and communication strategies must be evidence-based and tailored to specific populations. Providing clear, accurate information about vaccine safety and efficacy through trusted sources can help build confidence. Utilizing local community leaders and healthcare providers to disseminate pro-vaccine messages can be particularly effective in combating misinformation. Additionally, implementing convenient vaccination sites and offering incentives for vaccination can remove logistical obstacles and motivate individuals to get vaccinated [48,49].

The aVHS is a valuable tool for health professionals and public health authorities to identify and address vaccine concerns. For health professionals, the aVHS can pinpoint specific areas of VH, allowing for targeted conversations and interventions tailored to individual patient concerns. The aVHS provides data for public health to inform community-wide strategies to increase vaccine uptake by identifying common hesitancy patterns and tailoring public health campaigns accordingly. The most critical aspect of its usability is its ability to provide actionable insights that can be directly applied to improve vaccination rates and public health outcomes [48,49].

Health professionals can significantly benefit from understanding patients’ levels of dispositional optimism to address harmful thought patterns effectively. For both Greek and international audiences, incorporating assessments of optimism can help tailor communication strategies that foster a positive outlook toward vaccination. Optimistic individuals are more likely to engage in preventive health behaviors, including vaccination. By leveraging this trait, healthcare providers can frame vaccination positively, emphasizing its role in ensuring personal and communal well-being, thus reducing VH and promoting public health [48].

### 4.2. Study Limitations

This study had some inherent limitations. When interpreting this study’s conclusions, it is essential to consider the research participants’ geographical, demographic, socioeconomic, and cultural backgrounds. The convenience sampling strategy used is one of the protocol’s drawbacks. While convenience sampling may bring bias to the study results, it remains a viable strategy used in many investigations, particularly when a sample frame is not available. Moreover, recruiting all participants from a specific region of central Greece (Magnesia) may introduce selection bias. However, the researchers acknowledge that while some levels of selection bias are inevitable in this sampling process, biases related to gender and social desirability were not found to be significantly pronounced in our study. The demographic distribution of participants did not show substantial skewness towards any specific gender, and the nature of the questionnaire minimized the likelihood of socially desirable responses. Moreover, similar issues of social desirability bias are not reported in larger studies that utilized the same scales included in our questionnaire. Thus, while recognizing the limitations of our sampling approach, we believe the impact on gender representation and social desirability bias is relatively minimal.

Although the sample size was adequate for validating the aVHS, the convenience-sampling approach limits external validity and generalizability. The sample may not represent the broader population, and findings may not apply to other groups. Excluding those who did not receive any COVID-19 vaccine doses may also introduce selection bias. Additionally, convenience sampling can lead to unknown errors, and the reliability of self-reported data may increase bias and lead to unsafe conclusions. Exclusion criteria, such as cognitive impairment, documented history of mental illness, and refusal to provide informed consent, could further contribute to this bias.

## 5. Conclusions

This study demonstrated that the Greek version of the aVHS is valid and reliable. This research tool can be used in community settings using simple scoring algorithms; it can actively encourage vaccination patterns in upcoming infectious disease outbreaks and communicate the evidence required to create relevant policies or initiatives. Additionally, it will promote the development of vaccine research and eventually encourage proactive responses to newly emerging infectious diseases, improving the state of public health. Future research should use randomly sampled populations with different demographic features to achieve representative results about the psychometrics of this instrument.

## Figures and Tables

**Table 1:** Sample characteristics (*n* = 300).

Characteristics	Categories	*n*	*%*
Gender	Male	110	36.7
	Female	190	63.3
Age groups	20–29 years	84	28.0
	30–39 years	35	11.7
	40–49 years	50	16.7
	50–59 years	66	22.0
	≥60 years	65	21.7
Marital status	Single	110	36.7
	Married	150	50.0
	Divorced/widowed	40	13.3
Having children	No children	128	42.7
	Children aged 0–17 years	81	27.0
	Children aged ≥18 years	91	30.3
Place of residence	Urban (>10,000 inhabitants)	224	74.7
	Rural (<10,000 inhabitants)	76	25.3
Highest level of education	Tertiary (university)	162	54.0
	Secondary (intermediate or high school)	109	36.3
	Primary (elementary school)	29	9.7
Employment status	Employed (full or part-time)	174	58.0
	Unemployed	26	8.7
	Housewife/retired/student	100	33.3
Individual annual income (in EUR)	>20,000	20	6.7
	10,000–20,000	144	48.0
	<10,000	136	45.3
Self-perceived health status	Very good/good	229	76.3
	Fair	55	18.3
	Very poor/poor	16	5.3
**Characteristics**	**Mean ± Standard Deviation**	**Range**	**Cronbach’s Alpha**
Age (years)	44.87 ± 17.24	20–84	
Life Orientation Test-Revised	2.32 ± 0.64	0.33–4.00	0.715

**Table 2:** Distribution of answers to the 10 items of the adult Vaccine Hesitancy Scale (aVHS).

Items of aVHS	Strongly Disagree	Disagree	Neither Agree Nor Disagree	Agree	Strongly Agree
1. Vaccines are important for my health.*	1 (0.3%)	7 (2.3%)	45 (15.0%)	135 (45.0%)	112 (37.3%)
2. Vaccines are effective.*	0 (0.0%)	4 (1.3%)	57 (19.0%)	156 (52.0%)	83 (27.7%)
3. Being vaccinated is important for the health of others in my community.*	1 (0.3%)	5 (1.7%)	36 (12.0%)	152 (50.7%)	106 (35.3%)
4. All routine vaccinations recommended by the government are beneficial.*	4 (1.3%)	28 (9.3%)	77 (25.7%)	130 (43.3%)	61 (20.3%)
5. New vaccines carry more risks than older vaccines.	28 (9.3%)	91 (30.3%)	147 (49.0%)	27 (9.0%)	7 (2.3%)
6. The information I receive about vaccines from the government is reliable and trustworthy.*	4 (1.3%)	25 (8.3%)	114 (38.0%)	120 (40.0%)	37 (12.3%)
7. Getting vaccines is a good way to protect me from disease.*	1 (0.3%)	9 (3.0%)	47 (15.7%)	138 (46.0%)	105 (35.0%)
8. Generally, I do what my doctor or healthcare provider recommends about vaccines for me.*	0 (0.0%)	15 (5.0%)	54 (18.0%)	146 (48.7%)	85 (28.3%)
9. I am concerned about the serious adverse effects of vaccines.	10 (3.3%)	38 (12.7%)	84 (28.0%)	129 (43.0%)	39 (13.0%)
10. I do not need vaccines for diseases that are not common anymore.	35 (11.7%)	104 (34.7%)	89 (29.7%)	63 (21.0%)	9 (3.0%)

Notes: Data given as *n* (*%*). Five-point Likert scale was used (1 = strongly disagree to 5 = strongly agree). * Items with reverse scoring; aVHS: adult Vaccine Hesitancy Scale.

**Table 3:** Factor analysis of the adult Vaccine Hesitancy Scale (aVHS).

	EFA Loadings (*n* = 300)		CFA Standardized Regression Weights (*n* = 300)	
aVHS	aVHS Factor 1: Lack of Confidence	aVHS Factor 2: Risk Perception	aVHS Factor 1: Lack of Confidence	aVHS Factor 2: Risk Perception
Item 1	0.833	0.215	0.671	-
Item 2	0.857	0.169	0.624	-
Item 3	0.820	0.138	0.591	-
Item 4	0.799	0.178	0.716	-
Item 5	0.144	0.845	-	0.659
Item 6	0.692	0.219	0.562	-
Item 7	0.844	0.114	0.655	-
Item 8	0.818	0.099	0.631	-
Item 9	0.242	0.816	-	0.795
Item 10	0.106	0.790	-	0.651

aVHS: adult Vaccine Hesitancy Scale; EFA: exploratory factor analysis; CFA: confirmatory factor analysis.

**Table 4:** Internal consistency of the adult Vaccine Hesitancy Scale (aVHS).

aVHS	Mean ± *SD*	Range	Cronbach’s Alpha	Alpha If Item Deleted	Item-to-Total Correlation
Lack of Confidence	2.03 ± 0.67	1.00–4.29	0.919		
Item 1	1.83 ± 0.79	1.00–5.00		0.903	0.790
Item 2	1.94 ± 0.72	1.00–4.00		0.902	0.811
Item 3	1.81 ± 0.73	1.00–5.00		0.907	0.756
Item 4	2.28 ± 0.94	1.00–5.00		0.908	0.755
Item 6	2.46 ± 0.86	1.00–5.00		0.918	0.648
Item 7	1.88 ± 0.80	1.00–5.00		0.904	0.779
Item 8	2.00 ± 0.82	1.00–4.00		0.907	0.748
Risk Perception	2.94 ± 0.80	1.00–5.00	0.776		
Item 5	2.65 ± 0.86	1.00–5.00		0.671	0.648
Item 9	3.50 ± 0.98	1.00–5.00		0.665	0.642
Item 10	2.69 ± 1.03	1.00–5.00		0.761	0.561
Overall Scale	2.30 ± 0.60	1.00–4.00	0.884		

aVHS: adult Vaccine Hesitancy Scale; *SD*: standard deviation.

**Table 5:** Linear regression analyses with adult Vaccine Hesitancy Scale (aVHS) domains’ scores as dependent variables.

	Lack of Confidence				Risk Perception			
	Simple Regression		Multiple Regression		Simple Regression		Multiple Regression	
Independent Variable	*β* (*SE*)	*p*-Value	*β* (*SE*)	*p*-Value	*β* (*SE*)	*p*-Value	*β* (*SE*)	*p*-Value
Gender								
Male	0.00 ^a^				0.00 ^a^			
Female	−0.09 (0.08)	0.281			0.01 (0.10)	0.894		
Age (years)								
<45	0.00 ^a^				0.00 ^a^		0.00 ^a^	
≥45	0.01 (0.08)	0.984			0.33 (0.09)	<0.001	0.31 (0.09)	0.001
Marital status								
Single	0.00 ^a^				0.00 ^a^			
Married	−0.02 (0.08)	0.802			0.32 (0.10)	0.001		
Divorced/widowed	0.22 (0.12)	0.081			0.46 (0.14)	0.002		
Having children								
No children	0.00 ^a^				0.00 ^a^			
Children aged 0–17 years	0.04 (0.10)	0.696			0.15 (0.11)	0.173		
Children aged ≥18 years	−0.08 (0.09)	0.391			0.36 (0.11)	0.001		
Place of residence								
Urban	0.00 ^a^		0.00 ^a^		0.00 ^a^			
Rural	0.24 (0.09)	0.007	0.22 (0.09)	0.010	0.15 (0.11)	0.171		
Highest level of education								
Tertiary	0.00 ^a^				0.00 ^a^		0.00 ^a^	
Secondary	0.17 (0.08)	0.042			0.41 (0.09)	<0.001	0.41 (0.10)	<0.001
Primary	0.30 (0.13)	0.026			0.68 (0.15)	<0.001	0.58 (0.16)	<0.001
Employment status								
Employed	0.00 ^a^				0.00 ^a^		0.00 ^a^	
Unemployed	0.16 (0.14)	0.251			0.47 (0.17)	0.005	0.41 (0.16)	0.012
Housewife/retired/student	−0.07 (0.08)	0.423			0.18 (0.10)	0.064	−0.06 (0.10)	0.599
Individual annual income								
>EUR 20,000	0.00 ^a^		0.00 ^a^		0.00 ^a^			
EUR 10,000–20,000	0.44 (0.16)	0.005	0.39 (0.16)	0.012	0.35 (0.19)	0.065		
<EUR 10,000	0.55 (0.16)	<0.001	0.46 (0.16)	0.004	0.35 (0.19)	0.066		
Self-perceived health status								
Very good/good	0.00 ^a^				0.00 ^a^			
Fair	0.12 (0.10)	0.218			0.15 (0.12)	0.222		
Very poor/poor	0.09 (0.17)	0.610			0.07 (0.21)	0.753		
LOT-R score	−0.16 (0.06)	0.008	−0.12 (0.06)	0.047	−0.11 (0.07)	0.126		

^a^ Indicates reference category; *SE*: standard error; LOT-R: Life Orientation Test-Revised.

## Data Availability

Data are contained within the article.
